# Strand-specific RNA sequencing reveals extensive regulated long antisense transcripts that are conserved across yeast species

**DOI:** 10.1186/gb-2010-11-8-r87

**Published:** 2010-08-26

**Authors:** Moran Yassour, Jenna Pfiffner, Joshua Z Levin, Xian Adiconis, Andreas Gnirke, Chad Nusbaum, Dawn-Anne Thompson, Nir Friedman, Aviv Regev

**Affiliations:** 1 Broad Institute of MIT and Harvard, 7 Cambridge Center, Cambridge, MA 02142, USA; 2 Howard Hughes Medical Institute, Department of Biology, Massachusetts Institute of Technology, 31 Ames Street, 68-132, Cambridge, MA 02139, USA; 3 School of Engineering and Computer Science, Hebrew University, Ross Building, Givat Ram Campus, Jerusalem, 91904, Israel; 4 Alexander Silberman Institute of Life Sciences, Hebrew University, Edmond J Safra Campus, Givat Ram, Jerusalem, 91904, Israel

## Abstract

**Background:**

Recent studies in budding yeast have shown that antisense transcription occurs at many loci. However, the functional role of antisense transcripts has been demonstrated only in a few cases and it has been suggested that most antisense transcripts may result from promiscuous bi-directional transcription in a dense genome.

**Results:**

Here, we use strand-specific RNA sequencing to study anti-sense transcription in *Saccharomyces cerevisiae*. We detect 1,103 putative antisense transcripts expressed in mid-log phase growth, ranging from 39 short transcripts covering only the 3' UTR of sense genes to 145 long transcripts covering the entire sense open reading frame. Many of these antisense transcripts overlap sense genes that are repressed in mid-log phase and are important in stationary phase, stress response, or meiosis. We validate the differential regulation of 67 antisense transcripts and their sense targets in relevant conditions, including nutrient limitation and environmental stresses. Moreover, we show that several antisense transcripts and, in some cases, their differential expression have been conserved across five species of yeast spanning 150 million years of evolution. Divergence in the regulation of antisense transcripts to two respiratory genes coincides with the evolution of respiro-fermentation.

**Conclusions:**

Our work provides support for a global and conserved role for antisense transcription in yeast gene regulation.

## Background

Antisense transcription plays an important role in gene regulation from bacteria to humans. While the role of antisense transcripts is increasingly studied in metazoans [1], less is known about its relevance for gene regulation in the yeast *Saccharomyces cerevisiae*, a key model for eukaryotic gene regulation. Recent genomic studies using tiling microarrays showed evidence of stable antisense transcription in *S. cerevisiae *[2,3] and *Schizosaccharomyces pombe *[4,5].

It is unclear how broad the role of antisense transcription is and what key functional processes in yeast it affects. A few functional antisense transcripts have been implicated in the control of several key genes, including the meiosis regulator gene *IME4 *[6], the phosphate metabolism gene *PHO84 *[7], the galactose metabolism gene *GAL10 *[8], and the inositol phosphate biosynthetic gene *KCS1 *[9]. In contrast, genome-scale analysis in yeast suggested that antisense transcripts largely arise from bi-directional, possibly promiscuous, transcription from nucleosome free regions in promoters or 3′ UTRs of upstream protein coding genes [2,3]. The ability to massively sequence cDNA libraries (RNA-seq) can facilitate the discovery of novel transcripts [10-12], but most studies have not distinguished the transcribed strand.

Here, we used massively parallel sequencing to sequence a strand-specific cDNA library from RNA isolated from *S. cerevisiae *cells at mid-log phase. We found 1,103 putative antisense transcripts in those cells, ranging from short ones that cover only the 3′ UTR of sense genes to over a hundred long ones that cover the entire sense ORF. Many of the putative sense targets encode proteins with important roles in stationary phase, stress responses, or meiosis. We validated the differential regulation of 67 antisense transcripts and their sense targets in conditions ranging from nutrient limitation to stress, and show that the exosome component *Rrp6 *affects their levels, but that the histone deacetylase *Hda2 *does not. Furthermore, for a few examples we show that antisense transcripts and their differential regulation are conserved over 150 million years across five yeast species. Our results support a potential conserved role for antisense transcription in yeast gene regulation.

## Results

### Strand-specific RNA-seq of *S. cerevisiae *cells

To identify antisense transcripts in yeast, we used massively parallel sequencing (Illumina) to sequence a strand-specific cDNA library from *S. cerevisiae *during mid-log growth in rich media. The approach we used [13] relies on the incorporation of deoxy-UTP during the second strand synthesis, allowing subsequent selective destruction of this strand (Materials and methods). Our sequencing yielded 9.22 million 76-nucleotide paired-end reads that map to unique positions in the genome.

Of the reads that map to regions with a known annotation for uni-directional transcription (from the *Saccharomyces *Genome Database (SGD) [14]), only 0.62% were mapped to the opposite (antisense) strand, demonstrating the strand-specificity of our library [15] (Materials and methods). We next combined these reads to define consecutive regions of strand-specific transcription (Materials and methods), and found 8,778 units, covering 4,944 of the 5,501 (90%) genes expressed in this condition (top 85% [12]) at the correct orientation, for at least 80% of the length of each gene (Materials and methods; Additional files 1 and 2).

### Identification of 1,103 antisense transcripts that vary in sense coverage from the 3′ UTR to the entire ORF

We found 1,103 putative units that have an antisense orientation relative to annotated transcripts and cover at least 25% of a known transcript on the opposite strand, using published UTR estimates [2] (Materials and methods; Additional file 1). While antisense reads are only a small minority (0.62%) of the total reads, they aggregate in a relatively small number of loci, with 62% of the antisense reads concentrated in the 1,103 units we defined. The remaining 38% are mostly isolated reads scattered across the genome (Figure S1 in Additional file 3).

**Figure 1:**
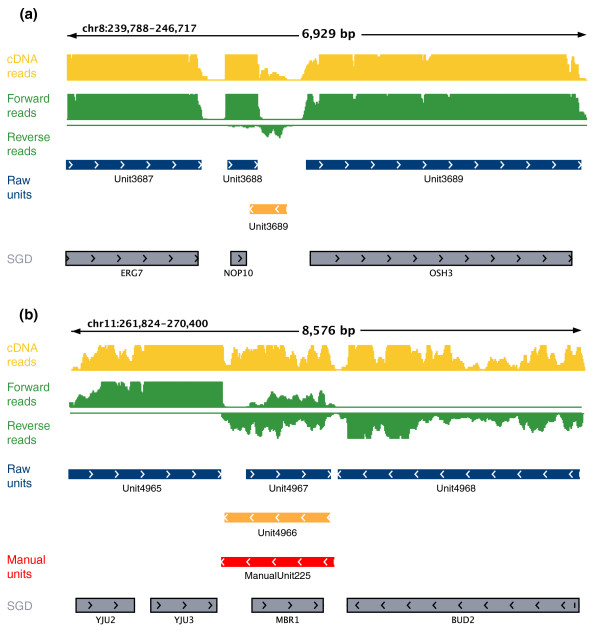
**Strand-specific RNA-seq identifies 1,103 antisense units associated with stationary phase, stress, and meiosis genes in *S. cerevisiae***. **(a)** Typical short antisense (*Unit3689*, antisense to *NOP10*). Shown are reads mapped from a standard cDNA sequencing library [15] (yellow), and from the strand-specific library prepared and run side-by-side on the same flow cell (green: forward reads above, reverse reads below). All coverage tracks were normalized to the total number of reads mapped, and are shown up to a threshold of 3 × 10^-8 ^of total mapped reads (genome-wide). Units were called from the strand-specific library (blue units, known genes; orange, putative antisense), and are shown along with the manually curated units (red) and the known gene annotations from the SGD (gray). **(b) **Typical long antisense (*ManualUnit225*, antisense to *MBR1*). Tracks are as in (a). The figures are shown using the Integrative Genome Viewer [36].

We observe a range of antisense unit lengths (Figure S2 in Additional file 3). At one extreme are 39 units that cover at least 25% of the transcript but none of the ORF, most commonly at the 3′ UTR (for example, *Unit3689*, a putative antisense transcript to *NOP10*; Figure 1a). Other units cover a substantial portion of the sense ORF. For example, 438 units overlap with at least 50% of the sense ORF, and 145 units cover the entire sense ORF (for example, *Unit4966*, a putative antisense to the *MBR1 *gene; Figure 1b). In some cases a single sense gene may be covered by more than one antisense unit, most likely due to low antisense expression levels that result in gaps in coverage (for example, *Unit8753*, *Unit8754*, *Unit8756 *and *Unit8758 *all opposite to the *OPT2 *gene; Figure S3 in Additional file 3). To avoid spurious or 'gapped' calls by our automatic method, we manually inspected each of the units, and focused on the 402 units that passed manual inspection and overlap at least 75% of a sense ORF (Materials and methods).

The 402 antisense units are supported by several lines of evidence. First, comparing the units to published data from strand-specific tiling arrays [2], we find that 143 of our 402 units (36%) are at least 80% covered by stable antisense units as previously defined [2], while 224 units were not detected at all on tiling arrays (Additional file 1; Materials and methods). Finally, 336 of the 402 units are supported by an independent RNA-seq experiment we conducted using an RNA ligation protocol [16] for strand-specific library preparation (Materials and methods) [15]. The lower number of units detected using the independent library reflects the less continuous nature of the data collected by the alternative protocol [15].

### Antisense units are unlikely to result solely from leaky transcription

We next assessed the previously suggested possibility [2,17] that antisense transcription is a consequence of leaky transcriptional regulation, through either unterminated transcription, bi-directional transcription initiation from promoters, or transcription from potential nucleosome-free regions (NFRs) in 3′ UTRs. We found that 48 and 27 units might reside within a long 3′ or 5′ UTR, respectively. Of the remaining 333 antisense units, 149 appear to share the (divergent) promoter of a known neighbor transcript, consistent with previous reports [2,3]. An additional 43 units may be transcribed from potential NFRs in the 3′ UTR of an adjacent transcript [18]. The remaining 141 units (35%) cannot be accounted for by transcription from a known promoter or 3′ UTR (when considering 400-bp margins; Figure S4 in Additional file 3).

We compared the change in expression of antisense units and such neighboring genes between cells grown in rich media containing glucose (yeast peptone dextrose (YPD)) and ethanol (yeast peptone ethanol (YPE)) as the main carbon source [2]. We reasoned that 'leaky transcription' would result in strong positive correlation in expression between the antisense transcript and the neighboring gene. However, we found a very low correlation (*R*^2 ^= 0.07; Figure S5 in Additional file 3), suggesting only weak co-regulation through leaky transcription, from divergent promoters or 3′ NFRs, if at all. Thus, even among the units that could hypothetically arise from leaky transcription, there is little if any evidence of such events.

We also examined the hypothesis that antisense is transcribed to prevent the neighboring gene from runthrough transcription. Of the 402 units, 72 (18%) end relatively close (< 200 bp) to the 3' ends of known genes (for example, *Unit3689 *ends close to the *NOP10 *gene shown in Figure 1a). On average, the 3′ UTRs of these 72 genes are shorter than those of other genes (*P *< 0.0058, Wilcoxon test; Figure S6 in Additional file 3). This minority of units could thus potentially play a role in curbing runthrough transcription.

### Stress, meiosis and nutrient limitation genes are associated with antisense transcripts at mid-log phase

To explore the potential function of the antisense units, we examined the known function and expression pattern of their associated sense transcripts. We found that the set of ORFs with 75% or more antisense coverage is enriched for genes induced after the diauxic shift (*P *< 6 × 10^-14^) or in stationary phase (*P *< 2 × 10^-10^), during stress (*P *< 2 × 10^-27^), and in some meiosis and sporulation experiments (for example, 85 of 805 genes induced at 8 h in a sporulation time course, *P *< 3 × 10^-6^), and include multiple central genes in these processes. For example, the genes encoding the key meiosis proteins *IME4*, *NDT80*, *REC102*, *GAS2*, *SPS19*, *SLZ1*, *RIM9*, and *SMK1 *are all associated with long antisense transcription. This is consistent with previous studies in *S. pombe *[4] showing a preponderance of antisense transcription in genes induced during meiosis. Long antisense is also found in many key respiration and mitochondrial genes, including *HAP3*, *COX8*, *CYB2*, *CYC3*, *COX5B*, *MMF1*, *NCA3*, *CYC1*, *MBR1*, *PET10*, *COX12*, and *ATP14*. Genes from other processes repressed during mid-log phase are also associated with long antisense transcripts. Notably, these include at least five members of the PHO regulon (*VTC1*, *PHO5*, *PHM8*, *ICS2*, *PHO3*) and three genes from the GAL regulon (*GAL4*, *GAL10*, *GAL2*). This suggests that antisense regulation may be prevalent across these regulons rather than at single target genes (as found in [6-8]). Furthermore, the expression of 149 of the antisense transcripts is inversely related to that of their sense targets, as measured on tiling arrays [2] in several conditions (glucose versus ethanol, versus galactose, and in Δ*rrp6*; Figure S7 in Additional file 3). Certain key genes that are highly expressed in mid-log phase are also associated with detectable transcription of long antisense units. These include some of the ribosomal protein genes (for example, *RPS26A*, *RPS20*), glycolytic enzymes (for example, *CDC19*, *PGK1*), and cell cycle regulators (for example, *PCL2*, *APC11*, *ASK1*). Nevertheless, these observations suggest that antisense transcription may be regulated in a condition-specific manner in *S. cerevisiae *and may be involved in the repression of stress, stationary phase and meiosis genes in rich growth conditions.

### Differential regulation of antisense-sense pairs in nutrient limitation and stress

**Figure 2:**
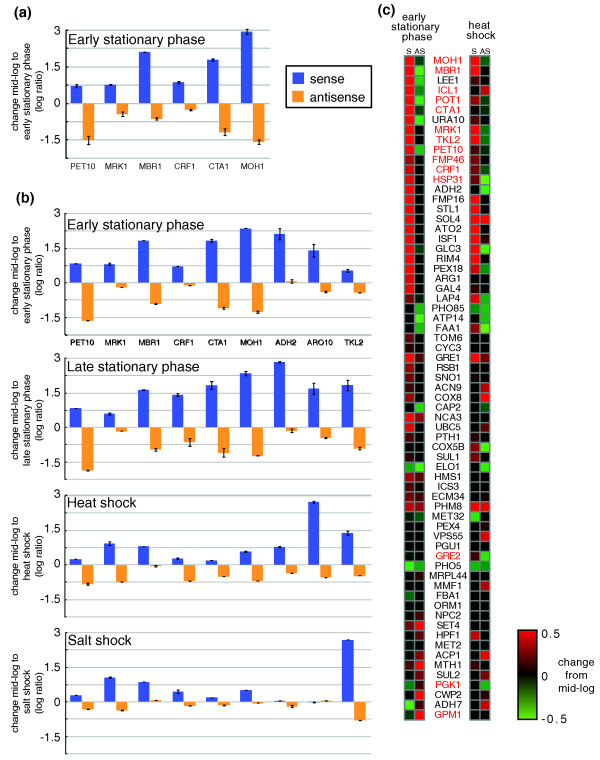
**Quantitative expression measurements of putative antisense units and the corresponding sense genes in *S. cerevisiae***. **(a) **Strand-specific qRT-PCR measurements of six pairs of known sense genes and their putative antisense units in comparing mid-log and early stationary phase (the y-axis shows the log_2 _ratio of expression in early stationary phase versus mid-log). Error bars indicate the standard deviation between biological replicates and different primers. **(b) **nCounter [20] measurements of nine representative putative antisense units, comparing mid-log to early stationary phase, stationary phase, heat shock and salt stress (the y-axis is as in (a) for the examined condition). Error bars indicate the standard deviation between biological replicates. (**c**) nCounter measurement for 67 tested sense-antisense pairs in early stationary phase (left) and heat shock (right), each relative to a mid-log (no stress) control. The columns marked 'S' and 'A' represent the sense and antisense change, respectively. Red, induced; green, repressed; black, no change. The names of genes highlighted in the main text are shown in red.

To test this hypothesis, we first experimentally measured the existence and differential expression of nine pairs of sense and antisense transcripts in *S. cerevisiae*, where the sense gene was known to be induced and important in stress or stationary phase states. We used strand-specific RT-PCR (Materials and methods) followed by sequencing to check for the presence of each sense and antisense transcript in mid-log (rich media), and found that all of the nine tested antisense units were present as expected (Additional file 4). Next, we used strand-specific quantitative real-time PCR (qRT-PCR; Materials and methods) to quantify the differential expression of six sense and antisense transcript pairs between mid-log and early stationary phase. We found that all six of the pairs were differentially expressed, with induction of the sense accompanied by repression of the antisense (Figure 2a; Additional file 5). Third, we devised a novel assay based on the nCounter technology for sensitive multiplex measurement of mRNAs [19,20] (Materials and methods) to measure the absolute level of expression of the nine pairs across five conditions, including mid-log, early stationary phase, stationary phase, high salt and heat shock. We found that the gene pairs exhibited inverse transcription patterns across all the tested conditions (Figure 2b). The differential expression we observed is consistent with antisense interference with sense expression (Figure 2b; Additional file 6), and with the known function and regulation of the sense genes. These included proteins with roles in respiration and mitochondria (*PET10 *and *MBR1 *[21,22]), repression of ribosomal protein gene expression in stress and poor nutrients (*CRF1 *[23]), and the response to caloric restriction (*CTA1 *[24]). Thus, differentially regulated antisense transcription may play a role in the distinction between mid-log non-stress growth and stationary phase and stress conditions in *S. cerevisiae*.

Finally, to test the generality of these suggestive patterns, we expanded the nCounter assay to measure the expression of 67 sense-antisense pairs in log-phase, early stationary phase, and after 15 minutes under heat shock conditions (Figure 2c; Additional file 6). We found 25 pairs where the sense was induced while the antisense was repressed in either early stationary phase or heat shock (12 in early stationary phase, 21 in heat shock, 8 in both), and 12 pairs where the sense was repressed while the antisense was induced (6 in early stationary phase, 8 in heat shock, 2 in both). Notably, 17 of the 25 pairs with induced sense and repressed antisense in early stationary phase (relative to mid-log) involved sense genes important in respiration, mitochondrial function, alternative carbon source metabolism and starvation response (for example, *PET10*, *MBR1*, *FMP46*, *POT1*, *MOH1*, *TKL2*, *ICL1*, *CTA1*). Conversely, four of the six pairs with the opposite pattern involved sense genes with key roles in glycolysis and fermentation (for example, *GPM1*, *PGK1*). Many of the pairs with induced sense and repressed antisense following heat shock overlapped with those responsive to early stationary phase (consistent with known metabolic changes under stress [25]). Furthermore, they also included four genes known to be important under environmental stresses (the regulators *CRF1 *and *MRK1*, and the effectors *HSP31 *and *GRE2*). Thus, antisense regulation may play a regulatory role at coordinating the major metabolic changes in the diauxic shift and early stationary phase, and some of the changes in the environmental stress response [21-24].

### The exosome component Rrp6 affects antisense levels, but the histone deacetylase Hda2 does not

To explore the mechanistic regulation of antisense transcription, we measured the expression of the 67 pairs of sense and antisense units using the nCounter assay in strains deleted for the exosome component RRP6 (Δ*rrp6*), the histone deacetylase HDA2 (Δ*hda2*), or both (Δ*rrp6*Δ*hda2*). Previous studies [2,7] have suggested that Δ*rrp6 *increases the levels of antisense transcription in the *PHO84 *locus, and that Hda2 is required for mediating the effect of antisense transcription on the sense transcripts in this locus. If these findings apply more broadly, we expect higher levels of antisense transcripts in Δ*rrp6*, and a change in the relative levels of sense to antisense in either the Δ*hda2 *or Δ*rrp6*Δ*hda2 *strains.

**Figure 3:**
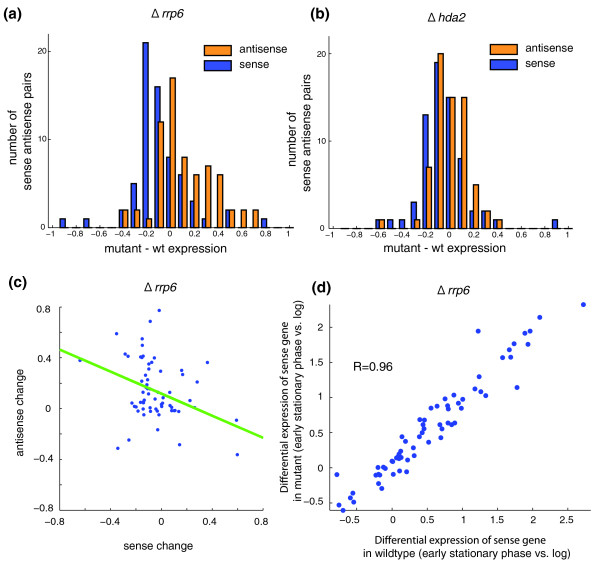
**Effect of Rrp6 and Hda2 on antisense transcript levels and sense-antisense regulation**. **(a,b)** The distribution of changes in expression levels (x-axis) for sense (blue) and antisense (orange) transcripts in the Δ*rrp6 *(a) and Δ*hda2 *(b) mutants compared to the wild type (wt). In the Δ*rrp6 *mutant (a) there is a mild increase in antisense levels and decrease in sense levels. No such changes are observed in the Δhda2 mutant (b). **(c)** Negative correlation between change in antisense transcript (y-axis) and in sense transcript (x-axis) in the Δ*rrp6 *mutant relative to the wild-type strain. **(d)** Similarity in differential sense gene expression from mid-log to early stationary phase between the wild type (x-axis) and the Δ*rrp6 *mutant (y-axis).

We found increased transcription of the antisense units in the Δ*rrp6 *mutant, with a mild reduction of the sense transcripts (*R *= -0.36; Figure 3a,c; Figure S8a in Additional file 3). This is consistent with regulation of antisense transcript levels by the exosome, and with a possible, albeit mild, effect of this increase in antisense on reduction in the level of sense transcripts. We found only a very mild, if any, effect on either sense or antisense transcripts levels in Δ*hda2 *(Figure 3b; Figure S8b in Additional file 3), suggesting that Hda2 plays at most a very minor independent role in the regulation of our transcripts. We also found no evidence for a synergistic effect between the mechanisms, since transcript levels in the double mutant were very close to those in Δ*rrp6 *(Figure S8c in Additional file 3). Finally, the differential expression of the sense genes between conditions was not substantially affected in any of these mutants (for example, *R *> 0.93 in all conditions; Figure 3d; Figure S9 in Additional file 3), suggesting that relative regulation itself was not compromised in any of these mutants. This may be due to a comparable effect of the deletion in all conditions. Thus, the mechanistic basis of sense-antisense regulation involved Rrp6, but may be more complex than that in the simple model suggested for *PHO84 *[7].

### Evolutionary conservation of six antisense transcripts and their regulation in five species of yeast

**Figure 4:**
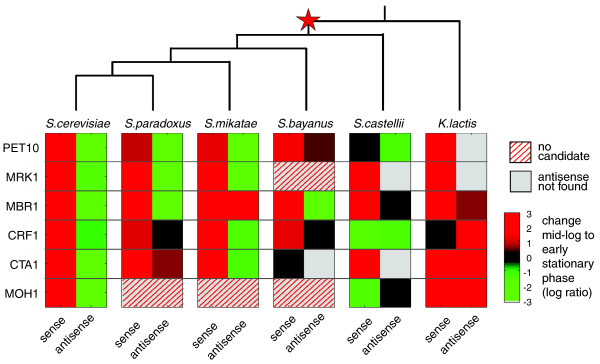
**Conservation of the presence and regulation of antisense units in *Hemiascomycota***. Shown are the differential expression values of antisense and sense units comparing mid-log and early stationary phase across *S. cerevisiae *and the five other species (red, higher in early stationary phase; green, lower in early stationary phase; black, no change; hatched, no candidate orthologous contig; grey, no antisense transcription detected in species). A phylogenetic tree of the species included in this study [27] is shown above (the star indicates the WGD).

Finally, we tested whether the presence and regulation of antisense transcripts is conserved in five other species of yeast. We reasoned that while the biochemical function and mechanistic basis of each antisense unit may be distinct or complex, their conservation would provide additional support for their functional and ancestral role in gene regulation. We chose five species with diverse lifestyles and a broad phylogenetic range spanning approximately 150 million years (Figure 4). These include three *sensu stricto Saccharomyces *species (*S. paradoxus*, *S. mikatae*, *S. bayanus*), a more distant species that diverged after the whole genome duplication (WGD; *S. castellii*), and one species that diverged pre-WGD (*Kluyveromyces lactis*). Importantly, post-WGD species are known to follow a respiro-fermentative lifestyle, repressing the expression of respiration genes (for example, *PET10*) in mid-log phase, whereas pre-WGD species follow a respirative lifestyle without such repression. We used conserved synteny and gene orthology of *S. cerevisiae *loci [26,27] to identify orthologous regions for candidate antisense transcription in the five species. We focused on six of the units validated in *S. cerevisiae *(*PET10*, *MRK1*, *MBR1*, *CRF1*, *CTA1*, *MOH1*), used strand-specific RT-PCR and sequencing to validate the presence of the orthologous sense and antisense transcripts in each species in mid-log and early stationary phase, and used strand-specific quantitative real-time PCR to quantify transcript levels (Additional file 5).

We found that the tested antisense units are largely conserved in the *sensu stricto *species, and less so at increasing evolutionary distances. All six units were detected in at least one species besides *S. cerevisiae*. Five of the six units are present in *sensu stricto Saccharomyces*, and four are still observed in *S. castellii *and *K. lactis*. The absence in *K. lactis *of an antisense transcript to the *PET10 *gene, important for respiratory growth, is consistent with its respiratory lifestyle, and suggests that antisense transcription in this gene may have appeared after the whole genome duplication. We cannot rule out the possibility, however, that other antisense units are present in the *K. lactis *genome, or that the missing antisense units are expressed under different conditions.

The anti-correlation between sense and antisense units observed in *S. cerevisiae *is conserved in most post-WGD species, but not in the pre-WGD *K. lactis*. The differential expression of five sense-antisense pairs (*PET10*, *MRK1*, *MBR1*, *CRF1*, *CTA1*) is conserved in at least two out of three other *sensu stricto *species. The more distant *S. castellii *shows less conservation of transcriptional regulation, most prominently in the *PET10 *gene. In contrast, although we could detect four of the antisense units in *K. lactis*, their differential expression was not conserved. This is consistent with the lack of repression of the corresponding sense gene in mid-log *K. lactis *cultures. The absence of antisense (for two genes) and the observed correlated (rather than anti-correlated) regulation (for three others) in *K. lactis *may reflect either the increased phylogenetic distance or may be more directly related to the shift to a respiro-fermentative lifestyle. In the latter case, either antisense transcription or its regulatory pattern in those genes may have evolved concomitantly with the emergence of fermentative growth, and the repression of respiratory genes, such as *PET10 *and *MBR1*. Further experiments are needed to elucidate this relationship.

## Discussion

In this study, we used strand-specific mRNA sequencing to explore the extent of antisense transcription in yeast, and found 1,103 putative antisense transcripts expressed in mid-log phase in *S. cerevisiae*, ranging from 39 short ones covering only the 3′ UTR of sense genes to 145 long ones covering the entire sense ORF. We focus on 402 long antisense units (each spanning over 75% of a coding unit). In this category, our sequencing based methodology allowed us to identify 224 new antisense transcripts that, in previous studies based on tiling microarrays [2], were either undetected or annotated as long UTRs of neighboring genes.

What could be the role of such prevalent antisense transcription? To date, functional studies have identified a regulatory role for a few antisense transcripts [6-8], whereas genome-wide analyses have suggested that antisense transcripts may represent promiscuous leaky transcription from NFRs at the promoter of a neighboring gene or the 3′ UTR of the sense gene [2,3,28]. The diversity of lengths in our 1,103 antisense units - ranging from long antisense units covering entire ORFs to shorter ones mostly at the 3′ UTR - suggests that there may be more than a single underlying mechanism for their formation and function.

Our results do not support promiscuous or aberrant transcription as the primary cause of the observed antisense transcripts. We find antisense transcription at only 18% of the genes. Moreover, many of the units are long and show robust sequence coverage, in contrast to what we might expect in a noisy process. Finally, antisense genes are only very weakly correlated to their neighbors, inconsistent with leaky transcription from divergent promoters or 3′ NFRs.

Characterizing the functional effect of each unit requires delicate assays to disable the antisense unit, without harming the sense gene, which have been successfully performed only in a few examples [6-8]. We therefore instead examined whether the changes in expression of sense and antisense are consistent with a regulatory function. We chose to focus on the long antisense units because they exhibit strong signal in our data, are less well-studied, are less likely to reflect noise, and can be verified more rigorously.

We found that the sense transcripts corresponding to longer antisense units are significantly enriched for key processes in *S. cerevisiae*, including stress response, the differential regulation of growth and stationary phase, and possibly meiosis and sporulation. The high level of antisense expression is consistent with the repression of these processes in fast growing yeast, and suggests a potential global function. Indeed, when we examined the relative change in expression in sense and antisense units across multiple conditions using three technologies (tiling arrays [2], strand-specific qPCR, and nCounter measurements), we found a strong and consistent anti-correlation between sense genes and the corresponding antisense units. While these results are consistent with regulatory function of antisense units (for example, reduction of antisense transcription leads to increased sense transcription), we cannot rule out the possibility that anti-correlation can occur without active regulation of the antisense transcript. For example, it is possible that when a sense gene is repressed, there is a relieved hindrance of antisense-transcription. Notably, we found support for the role of Rrp6 in the regulation of antisense levels, resulting in an increase in antisense levels in the Δ*rrp6 *mutant, and a concomitant, albeit very mild, decrease in sense levels. We could not demonstrate a general effect of Hda2 on the levels of sense or antisense transcripts (either alone or together with Rrp6), and - in all mutants - the differential expression of sense and antisense remained highly correlated to the wild-type regulation. This suggests that it may be challenging to generalize the mechanisms shown for specific transcripts (*PHO84*) to all antisense transcripts.

Independent support for a potential function is the conservation of expression and regulation of six antisense units tested across five species that have diverged more than 150 million years ago, suggesting purifying selection. Notably, previous studies in mammals have shown that certain non-coding RNAs (that are not antisense) can be conserved at the sequence level [17,29], but the applicability of such analyses to antisense transcripts that cover ORFs is limited, and hence experimental data are needed to show conservation. We find that both the presence and the regulation of antisense transcripts are most diverged in the distant, pre-WGD species *K. lactis*. This may reflect either the increased phylogenetic distance *per se*, or an evolved role in regulating respiration genes in post-WGD species. Another possibility for the lack of conservation in expression or absence of antisense in *S. castellii *and *K. lactis *may be the presence of RNA interference in these species [30]. Further experiments will be needed to elucidate these possibilities and characterize the full functional scope of antisense transcription in yeasts.

## Conclusions

Our results expand and strengthen the existing body of evidence that antisense transcription is a substantial phenomenon in yeast, and not solely a noisy by product of imprecise transcription regulation. While the mechanism and function of antisense transcription is still elusive, our results indicate that antisense transcription is often conserved and plays a regulatory role in the yeast transcriptional response.

## Materials and methods

### Supplementary website

All tables, figures, raw sequenced reads, and a link to a browser with the mapped reads appear on our supplementary website [31].

### Strains and growth conditions

Strains are listed in Table 1. Cultures were grown in the following rich medium: yeast extract (1.5%), peptone (1%), dextrose (2%), SC Amino Acid mix (Sunrise Science - San Diego, CA, USA) 2 g/l, adenine 100 mg/l, tryptophan 100 mg/l, uracil 100 mg/l, at 200 RPM in a New Brunswick Scientific (Edison, NJ, USA) air-shaker. The medium was chosen to minimize cross-species variation in growth. Following the experimental treatments described below, stressed and mock cultures were transferred to shaking water baths.

**Table 1:** Strains and growth conditions

Strain number	Species	Background	Genotype	Source
BB32	*Saccharomyces cerevisiae*			Gift from Leonid Kruglyak's lab
BY4741	*Saccharomyces cerevisiae*	S288c	MATa, his3Δ1, leu2Δ0, met15Δ0, ura3Δ0	Gift from Andrew Murray's lab
	*Saccharomyces cerevisiae*	BY4741	Same as above with rrp6Δ::KANMX6	ATCC
	*Saccharomyces cerevisiae*	BY4741	Same as above with hda2Δ::URA3	Gift from Oliver Rando's lab
	*Saccharomyces cerevisiae*	BY4741	Same as above with rrp6Δ::KANMX6, hda2Δ::NatMX4	This study
NCYC2600	*Saccharomyces paradoxus*			NCYC Stock Center
IFO 1815	*Saccharomyces mikatae*			ATCC
CLIB 592	*Saccharomyces castellii*			CLIB Stock Center
CLIB 209	*Kluyveromyces lactis*			CLIB Stock Center

ATCC, American Type Culture Collection.

To generate strain RGV 69(*rrp6Δ::KANMX6*, *hda2Δ::NatMX4*), strain RGV 71(*rrp6Δ::KANMX6*) was transformed with a PCR product constructed by using the pAG25 containing the NatMX4 cassette using the following primers: GTAAAAGTATTTGGCTTCATTAGTGTGTGAAAAATAAAGAAAATAGATACAATACTATCGACGGTCGACGGATCCCCGGGTT and AAGAAAGTATATAAAATCTCTCTATATTATACAGGCTACTTCTTTTAGGAAACGTCACATCGATGAATTCGAGCTCGTT [32]. Correct integration of this construct was confirmed with the following: (5′ left) left TGGCGTATATGGTTCATTGC; (5′ right) GTATGGGCTAAATGTACGGG; (3′ left) left TGGCGTATATGGTTCATTGC; (3′ right) GGTTGGAGAGGCAAATTGAG.

### Heat shock

Overnight cultures of *S. cerevisiae *were grown in 650 ml of media at 22°C to between 3 × 10^7 ^and 1 × 10^8 ^cell/ml, OD_600 _= 1.0. The overnight culture was split into two 300 ml cultures and cells from each were collected by removing the media via vacuum filtration (Millipore - Billerica, MA, USA). The cell-containing filters were re-suspended in pre-warmed media to either control (22°C) or heat-shock temperatures (37°C). Density measurements were taken approximately 1 minute after cells were re-suspended to ensure that concentrations did not change during the transfer from overnight media. We harvested 12 ml of culture at 15 minutes and quenched by adding to 30 ml liquid methanol at -40°C, which was later removed by centrifugation at -9°C, and stored these overnight at -80°C. Cell density measurements were repeatedly taken every 5 to 15 minutes for the first 2 hours after treatment. Harvested cells were later washed in RNase-free water and archived in RNAlater (Ambion - Austin, TX, USA) for future preparations. Cells were also harvested from cultures just before treatment for use as controls.

### Salt stress

Overnight cultures of *S. cerevisiae *(*BB32*) were grown in 600 ml of media at 30°C until reaching a final concentration of 3 × 10^7 ^and 1 × 10^8 ^cell/ml. The culture was split into two parallel cultures of 250 ml and sodium chloride was added to one culture for a final concentration of 0.3 M NaCl. Cells were harvested by vacuum filtration at 15 minutes after the addition of sodium chloride and from cultures immediately before the addition of sodium chloride for use as controls (t = 0 minutes). Filters were placed in liquid nitrogen and stored at -80°C and were later archived in RNAlater for future use.

### Diauxic shift

Overnight cultures for each species were grown to saturation in 3 ml rich medium. From the 3 ml overnight cultures, 300 ml of rich media was inoculated at the OD_600 _corresponding to 1 × 10^6 ^cell/ml: *S. cerevisiae *0.016, *S. paradoxus *0.016, *S. mikatae *0.023, *S. bayanus *0.016, *S. castellii *0.020, and *K. lactis *0.024. The density measurements were taken approximately 1 minute after cells were re-suspended to ensure that concentrations did not change during the transfer from overnight media. Cells were harvested and quenched at a final concentration of 60% methanol at the mid-log and early stationary phase time points. Mid-log was taken at the following OD_600 _values: *S. cerevisiae*, 0.35; *S. paradoxus*, 0.40; *S. mikatae*, 0.40; *S. bayanus*, 0.30; *S. castellii*, 0.35; and *K. lactis*, 0.30. The early stationary phase time points were taken 2 hours after the glucose levels reached zero. Glucose levels were monitored hourly using the YSI 2700 Select Bioanalyzer (YSI Life Sciences - Yellow Springs, OH, USA). OD_600 _values for early stationary phase time points were: *S. cerevisiae*, 4.6; *S. paradoxus*, 3.9; *S. mikatae*, 4.3; *S. bayanus*, 2.8; *S. castellii*, 3.2; and *K. lactis*, 5.0. Harvested cells were later washed in RNase-free water, archived in RNAlater (Ambion) for future preparations, and frozen at -80°C.

### Stationary phase

Stationary phase was done for *S. cerevisiae *(*BB32*) only. This experiment was set up identically to the diauxic shift, but samples were taken at mid-log, and 5-day time points. The 5-day samples were taken at the same time of day as the mid-log samples.

### Strand-specific cDNA library

The library was created by modifying the previously described dUTP second strand method [13]. All reagents were from Invitrogen (Carlsbad, CA, USA) except as noted. We fragmented 200 ng of *S. cerevisiae *polyA^+ ^RNA by heating at 98°C for 40 minutes in 0.2 mM sodium citrate, pH 6.4 (Ambion). Fragmented RNA was concentrated to 5 μl, mixed with 3 μg random hexamers, incubated at 70°C for 10 minutes, and placed on ice. First-strand cDNA was synthesized with this RNA primer mix by adding 4 μl of 5× first-strand buffer, 2 μl of 100 mM DTT, 1 μl of 10 mM dNTPs, 4 μg of actinomycin D (USB), 200 U SuperScript III, and 20 U SUPERase-In (Ambion) and incubating at room temperature for 10 minutes followed by 1 hour at 55°C. First-strand cDNA was cleaned up by extraction twice with phenol:chloroform:isoamyl alcohol (25:24:1), followed by ethanol precipitation with 0.1 volumes 5 M ammonia acetate to remove dNTPs and re-suspension in 104 μl H_2_O. Second-strand cDNA was synthesized by adding 4 μl 5× first-strand buffer, 2 μl 100 mM DTT, 4 μl 10 mM dNTPs with dTTP replaced by dUTP (Sigma - Aldrich, St Louis, MO, USA), 30 μl 5× second strand buffer, 40 U *Escherichia coli *DNA polymerase, 10 U *E. coli *DNA ligase, 2 U *E. coli *RNase H and incubating at 16°C for 2 hours. A paired-end library for Illumina sequencing was prepared according to the instructions provided with the following modifications. First, five times less adapter mix was ligated to the cDNAs. Second, 1 U USER (New England Biolabs - Ipswich, MA, USA) was incubated with 180- to 480-bp size-selected, adapter-ligated cDNA at 37°C for 15 minutes followed by 5 minutes at 95°C before PCR. Third, PCR was performed with Phusion High-Fidelity DNA Polymerase with GC buffer (New England Biolabs) and 2 M betaine (Sigma). Fourth, PCR primers were removed using 1.8× volume of AMPure PCR Purification kit (Beckman Coulter Genomics - Danvers, MA, USA).

### Strand-specific library based on the RNA ligation method

The RNA ligation library was created using a previously described method [16] starting from 1.2 μg of polyA^+ ^RNA with the following modifications. RNA was fragmented by incubation at 70°C for 8 minutes in 1× fragmentation buffer (Ambion) and 65- to 80-nucleotide RNA fragments were isolated from a gel. RNA was reverse transcribed with SuperScript III (Invitrogen) at 55°C and cDNA was amplified with Herculase (Agilent - Santa Clara, CA, USA) in the presence of 5% DMSO for 16 cycles of PCR followed by a clean up with 1.8× volumes of AMPure beads (Beckman Coulter Genomics - Danvers, MA, USA) rather than gel purification.

### Illumina sequencing

Both cDNA libraries were sequenced with an Illumina Genome Analyzer II (San Diego, CA, USA). The dUTP library was sequenced using 1 lane of 76-nucleotide paired reads, and the RNA ligation library was sequenced using 2 lanes of 51-nucleotide reads. All RNA-seq data are available in the Gene Expression Omnibus [GEO:GSE21739].

### Data pre-processing

We used the Arachne mapper [33] to map the reads to the genome. We next identified consecutive regions of transcription by segmenting the centers of the paired-end segments with coverage >1 and maximum signal gaps of size 20 nucleotides.

### Assessment of the strand specificity of the library

To evaluate the strand specificity of our library, we used the known annotation from SGD [14], and published estimates of UTR lengths [2], or when absent an estimation of 100 bp. According to these annotations we found that only 53,803 reads (0.62%) mapped to the opposite strand of known transcripts.

### Identification of sense and antisense transcriptional units

We assigned a putative unit to a known gene if it is in the same orientation as the unit and it overlaps the known transcript boundaries, including published estimates of UTR length [2], or when absent an estimation of 100 bp was used. When comparing our transcription units to known annotations in the SGD [14], we examined the top 85% of expressed genes, as previously described [12].

### Manual annotation of 402 antisense units

We have manually annotated the boundaries of antisense units covering 75% or more of an opposite ORF, resulting in 402 antisense units covering 75% or more of 412 ORFs.

### Comparing the antisense units to published data from strand-specific tiling arrays

We compared our units to the published catalog of [2] using the following criteria. For each of our units, we searched for units in the catalog of [2] that are on the same strand and overlap it. We chose the unit with the highest overlap, and required a minimal threshold of 50% overlap.

### Functional analysis of sense units

We constructed a gene set from the 377 sense genes, for which at least 75% of the ORF is covered by an antisense unit, and tested it for functional enrichment using a collection of functional categories as previously described [27]. We also tested the genes for enriched induction or repression in a compendium of 1,400 annotated arrays, as previously described [27].

### Identification of candidate regions in other species

We searched for orthologs of the sense gene in other species, using our published orthogroup catalog [27], and used the relative coordinates of the antisense transcripts in *S. cerevisiae *relative to the sense gene to predict their locations in other species. In cases where there were no clear candidates for orthologs, or the synteny block was broken [26], we did not define a candidate.

### Strand-specific RT-PCR

Strand-specific RT-PCR followed an adaptation of a published protocol [34]. Total RNA was isolated from strain Bb32(3) at late log time point for two biological replicates. RNA was Turbo DNase treated (Ambion) following the manufacturer's stringent protocol followed by phenol chloroform extraction. For each assay, a gene-specific, strand-specific reverse transcription (RT) was performed. The four reactions for each sample were: +RT L-primer (sense), +RT R-Primer (antisense), +RT no primer, -RT both primers. First strand cDNA synthesis started RNA denaturation and the hybridization of the 2 pmol of gene specific primer. Total RNA with primer (10 ng) was heated to 70°C for 10 minutes and incubated on ice for at least 1 minute. A primer targeting ACT1 mRNA was always included as an internal control for strand specificity. This was followed by adding a Master mix containing 200 U SuperScript III (Invitrogen), 40 U RNaseOut (Invitrogen) and 10 mM dNTP mix for at 55°C for 15 minutes. The enzyme was heat-inactivated at 70°C for 15 minutes. RNA complementary to the cDNA was removed by *E. coli *RNase H (10 U; Ambion) and remaining RNAs were digested with 20 U of RNase Cocktail (Ambion) by incubating at 37°C for 20 minutes. PCR was performed for the sense and antisense transcripts independently. We added 5 μl of RT to each reaction as template with two gene-specific primers each at 250 nM final concentration (the same primers that were used for the sense and antisense RT; Additional file 7), 300 μM dNTP and 1 U of Ampli Taq Gold (Applied Biosystems - Carlsbad, CA, USA), in a 50 μl reaction. RNA contaminated with genomic DNA was used as a positive control. The touch down amplification program used was as follows: incubation of 95°C for 5 minutes followed by 10 cycles of 95°C for 30 s, 60°C for 30 s -1 degree per cycle, 70°C for 45 s, then followed by 17 to 20 cycles of 95°C for 30 s, 50°C for 30 s, 70°C 45 s, 72°C for 10 minutes (a step required for future Topo TA cloning (Invitrogen)).

### Strand-specific RT-PCR across species

Strand-specific RT-PCR across species used an adaptation of a published protocol [35]. Total RNA was isolated from each species at both the mid-log and early stationary phase time points. Genomic DNA contamination was removed with Turbo DNase (Ambion) using the stringent protocol, and phenol:chloroform to extract the RNA and to inactivate the DNase. For each of the species two biological replicates of the mid-log and early stationary phase time points were tested. Four reactions were performed for each sample: +RT L-primer (sense), +RT R-primer (antisense), +RT no primer, -RT. The sense, antisense, and -RT reactions were done with 2 pmol of primer (Additional file 7; only the primers with A1 in the title were used for the initial RT-PCR, and all primers used were designed for the target species). RT was done with first strand synthesis only in 20-μl reactions, using 4 units of Omniscript reverse transcriptase (Qiagen - Valencia, CA, USA) and 500 ng of total RNA. Each reaction was carried out at 50°C for 20 minutes, and heat inactivated at 70°C for 15 minutes. PCR was conducted as for the *S. cerevisiae *RT-PCR described above.

### Strand-specific qRT-PCR across species

The same RT protocol was followed for the qRT-PCR across species as for the RT PCR above. For each sense-antisense pair validated, two sets of primers were tested, and primers for two internal control genes (*ACT1 *and *PDA1*) were included in each reaction. Control primers ('right primer', Additional file 7) were added at a concentration of 2 pmol to each of the RT reactions. qPCR was done using the Roche Light Cycler 480 in 12-μl reactions in a 384 well plate (Roche - Indianapolis, IN, USA). qPCR was done independently for sense, antisense, and control genes. RT samples were diluted 1:40 in water then 1:2 in Light Cycler 480 SYBR Green I Master with gene specific primer pair (each primer at 200 nM final concentration). The program protocol used was as follows: activation, 95°C for 5 minutes; cycling, 95°C for 15 s and 60°C for 45 s; melt, 95°C continuous.

### Analysis of strand-specific qRT-PCR data

The ratios reported in Additional file 5 and Figure 2a are log_2 _ratios of early stationary phase and mid-log qRT-PCR reads (after normalization by the control gene *PDA1*), averaged over the two sets of primers and the two biological repeats.

### nCounter measurements

The following experiments were done in biological duplicates: heat shock - 0 and 15 minutes; salt stress - 0 and 15 minutes; diauxic shift - log and early stationary phase; and stationary phase - log and 5 days. Details on the nCounter system are presented in full in [20]. In a nutshell, the nCounter system uses pre-defined probes labeled with molecular barcodes ('code sets') and single molecule imaging to detect and directly count millions of unique transcripts (from up to hundreds of genes) in a single reaction. The assay is performed in cell lysates, involves no enzymatic steps prior to detection, and is highly accurate. Code sets were constructed to detect putative antisense units and sense genes and additional controls (Additional file 8). We lysed 7 × 10^7 ^(or 2 × 10^7^, depending on the code set) cells according to the RNeasy (Qiagen) yeast mechanical lysis protocol. The protocol was stopped after spinning the lysate to remove debris, and 3 μl of the lysate was hybridized for 16 hours followed by processing in the nCounter Prep Station and quantification by the nCounter Digital Analyzer. We normalized the nCounter data in two steps as previously described [19]. In the first step, we controlled for small variations in the efficiency of the automated sample processing. To this end, we followed the manufacturer's instructions, and normalized measurements from all samples analyzed on a given run to the levels of a chosen sample (in all cases we used the first sample in the set). This was done using the positive spiked-in controls provided by the nCounter instrument. In the second step, we used the control genes for which we designed probes to normalize for sample variation.

## Abbreviations

bp: base pair; DTT: dithiothreitol; NFR: nucleosome-free region; ORF: open reading frame; qRT-PCR: quantitative reverse transcriptase PCR; RNA-seq: RNA sequencing; SGD: *Saccharomyces *Genome Database; UTR: untranslated region; WGD: whole genome duplication; YPD: yeast peptone dextrose; YPE: yeast peptone ethanol.

## Authors' contributions

MY, JP, JZL, AG, CN, D-AT, NF, and AR designed the research; MY, JP, JZL, XA, D-AT, NF, and AR performed research; MY, NF, and AR analyzed data; JZL and XA contributed text to the methods section; and MY, NF, and AR wrote the paper with editorial input from all authors. All authors read and approved the final manuscript.

## Supplementary Material

Additional file 1 **Table S1**. Strand-specific (sense and antisense) transcribed units in mid-log *S. cerevisiae*. Click here for file

Additional file 2 **Table S2**. Sense and antisense coverage of SGD annotated genes. Click here for file

Additional file 3 **Figure S1 to S9**. Figure S1: read coverage at antisense units. **(a,b) **The distribution (a) and cumulative distribution **(**CDF) (b) of read coverage at antisense units 'called' by our method (gray) and at all other loci in the genome with at least one antisense read (orange). The called units have substantially deeper coverage, whereas 80% of sporadic loci are covered by a single read. (**c**) Sense coverage (x-axis) versus antisense coverage (y-axis) of all verified genes. Genes that we have detected antisense units opposite them are shown in orange. Figure S2: statistics for transcription units. (**a**) Distribution of antisense unit length, colored by the percentage of overlap with the opposite ORF. Dark blue, units with at least 25% overlap with the opposite transcript; light blue, units with at least 50% overlap with the opposite ORF; green, units with at least 75% overlap with the opposite ORF; orange, units with 100% overlap with the opposite ORF. (**b**) Cumulative distribution function of the units length. Blue, antisense units; red, other units. Figure S3: an example of an over-segmented antisense unit. Shown is the genomic region of *OPT2*; tracks and colors are as in Figure 1, with the addition of the brown tracks showing the centers of the paired end segments (forward and reverse), which were used for the segmentation (Materials and methods). All coverage tracks are normalized and shown up to a threshold of 3 × 10^-8 ^of the total (genome-wide) number of mapped reads. Due to low read coverage, both the sense (blue) and the antisense units (yellow) are over-segmented. After the manual curation of the antisense units, we defined one long antisense unit (*ManualUnit402*) that covers the entire ORF of the gene *OPT2*. The figure is shown using the Integrative Genome Viewer [36]. Figure S4: promoter types associated with antisense units. Shown are two examples of promoter types of antisense units; tracks and colors as in Figure 1. *ManualUnit69 *included the *BTT1 *gene, and a very long 3′ UTR, as an antisense to the gene *MET32*. *ManualUnit70 *is a long antisense to the gene *CTA1*, and is transcribed from the divergent promoter of *RMD5*. The figures are shown using the Integrative Genome Viewer [36]. Figure S5: correlation between differential expression of antisense units and their neighboring (non-overlapping) genes. Expression of antisense units versus neighboring genes, which could be co-regulated (using published tiling array data [2]). Shown is the log ratio of change from glucose (YPD) to ethanol (YPE). Blue, antisense units with shared promoter (as in Figure S3 in Additional file 3); red, antisense units with a nearby 3′ UTR; green, linear fit. Figure S6: differences in UTR length between genes with nearby antisense units, compared to all genes. Cumulative distribution of the UTR lengths of all genes (blue) and those with antisense units ending close to the 3′ UTR end. Figure S7: differential expression of antisense units and their target sense transcripts. (**a**) Expression of sense versus antisense units (using published tiling array data [2]). Shown is the log ratio of change in sense gene expression from YPD to YPE (x-axis) plotted versus the same for the antisense strand (y-axis). Red, differentially expressed genes; green, linear fit. (**b,c**) The same as (a), only comparing YPD to galactose growth and to an rrp6 deletion mutant, respectively. Figure S8: mutant effect on transcription. **(a-c) **Expression changes of the sense genes (x-axis) versus expression changes of the antisense units (y-axis) in the Δ*rrp6 *mutant (a), the Δ*hda2 *mutant (b), and the Δ*rrp6*Δ*hda2 *mutant (c). Figure S9: mutant effect on differential expression. **(a-c) **Differential expression of the sense genes from mid-log to early stationary phase in the wild type (x-axis) versus the Δ*rrp6 *mutant (a), the Δ*hda2 *mutant (b), and the Δ*rrp6*Δ*hda2 *mutant (c). Click here for file

Additional file 4 **Table S3**. Antisense units validated in RT experiments in *S. cerevisiae*. Click here for file

Additional file 5 **Table S4**. qRT-PCR results in each gene and species. Click here for file

Additional file 6 **Table S5**. Nanostring results in *S. cerevisiae*. Click here for file

Additional file 7 **Table S6**. RT and qRT-PCR primers in each gene and species. Click here for file

Additional file 8 **Table S7**. Control genes used for the Nanostring nCounter assays. Click here for file
