# Supplementary material for: Strand-specific RNA sequencing reveals extensive regulated long antisense transcripts that are conserved across yeast species
Source: Genome Biol. 2010 Aug 26;11(8):R87. doi: 10.1186/gb-2010-11-8-r87 (PMC2945789; doi:10.1186/gb-2010-11-8-r87)

Supplementary Figure 1 - Antisense reads coverage: units vs. sporadic

a Read coverage histograms

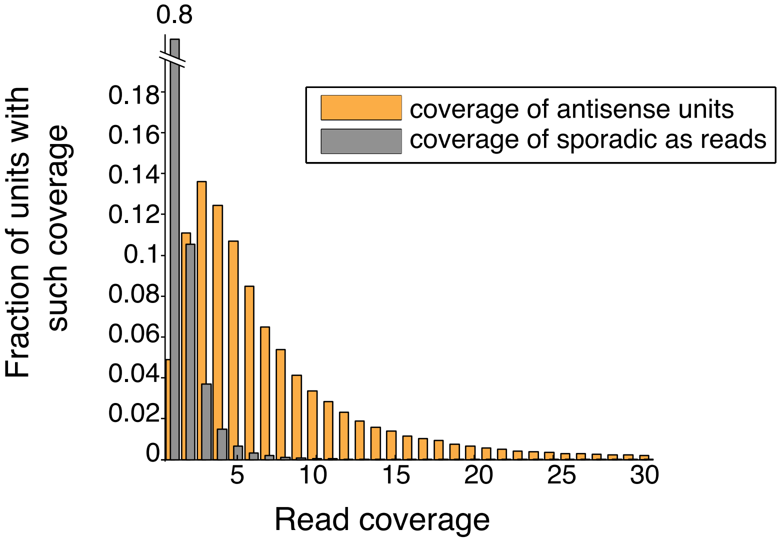

b Empirical CDF

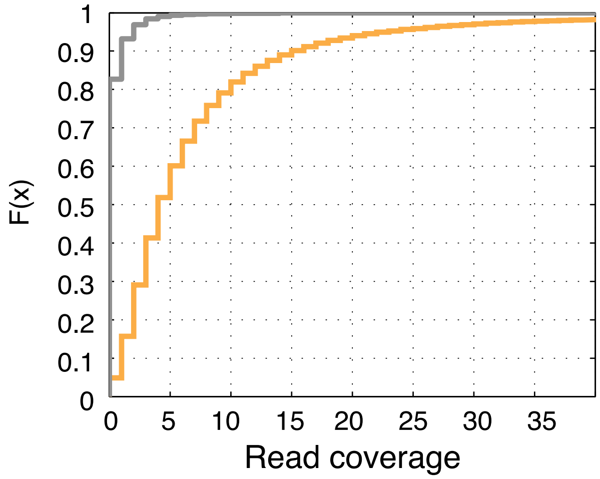

c Sense coverage vs. antisense coverage of genes

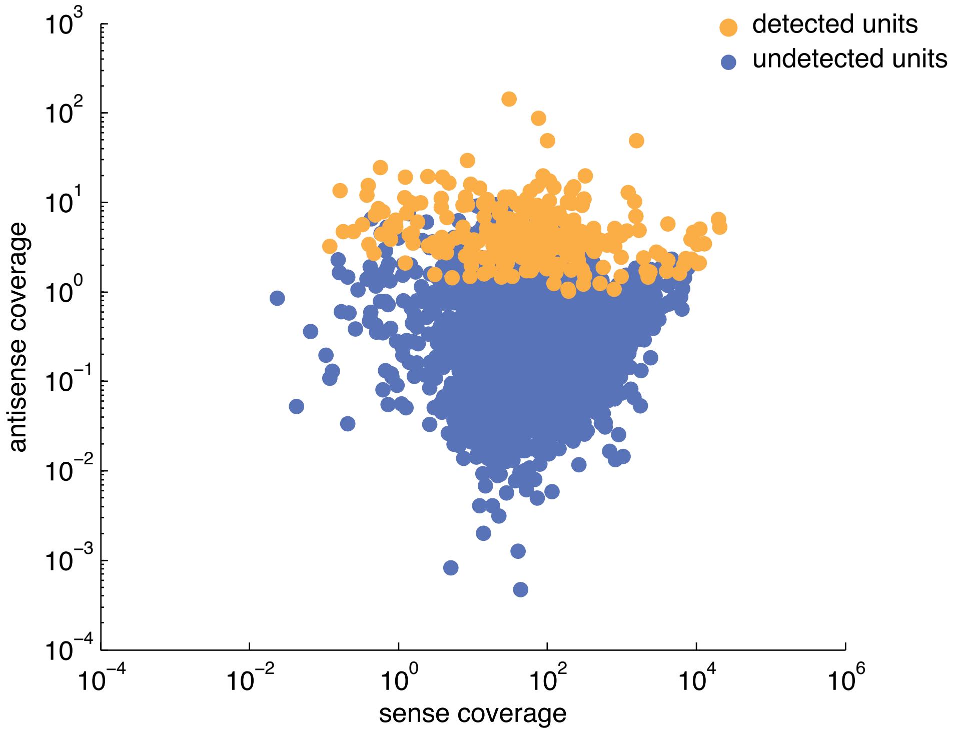

Supplementary Figure 2 - Units statistics

a Antisense unit length histogram

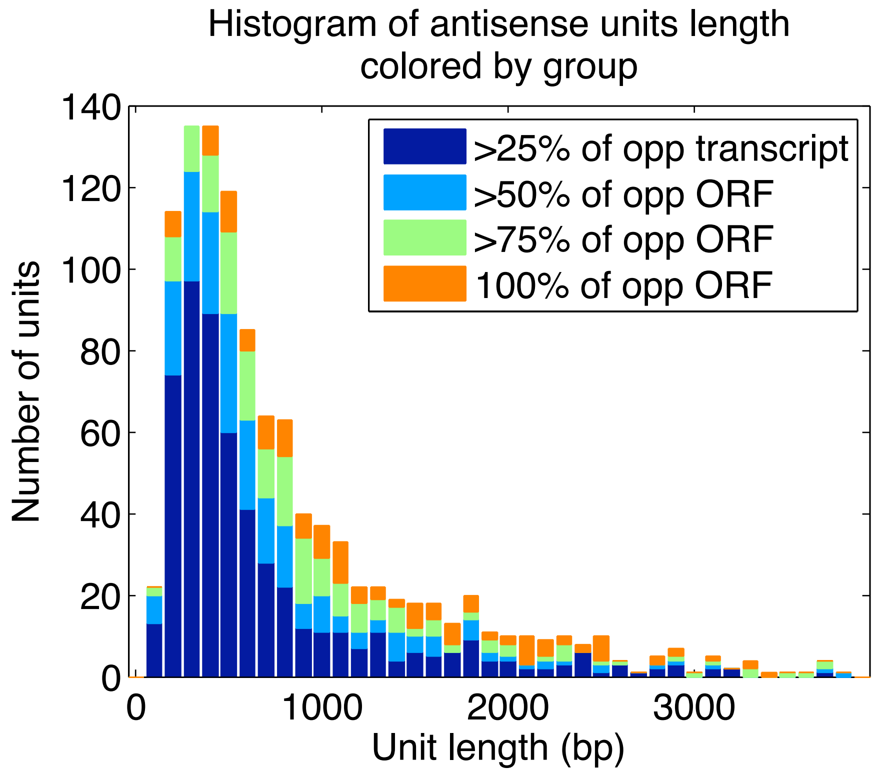

b Cumulative distribution function (cdf) of antisense units vs. other units

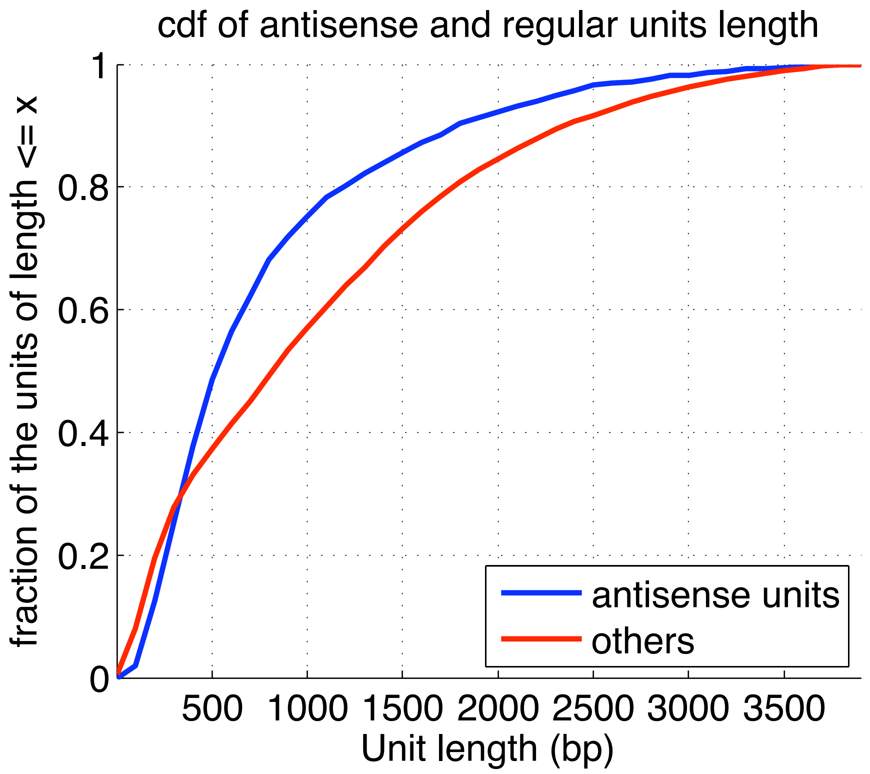

Supplementary Figure 3 - Manual Curation Example

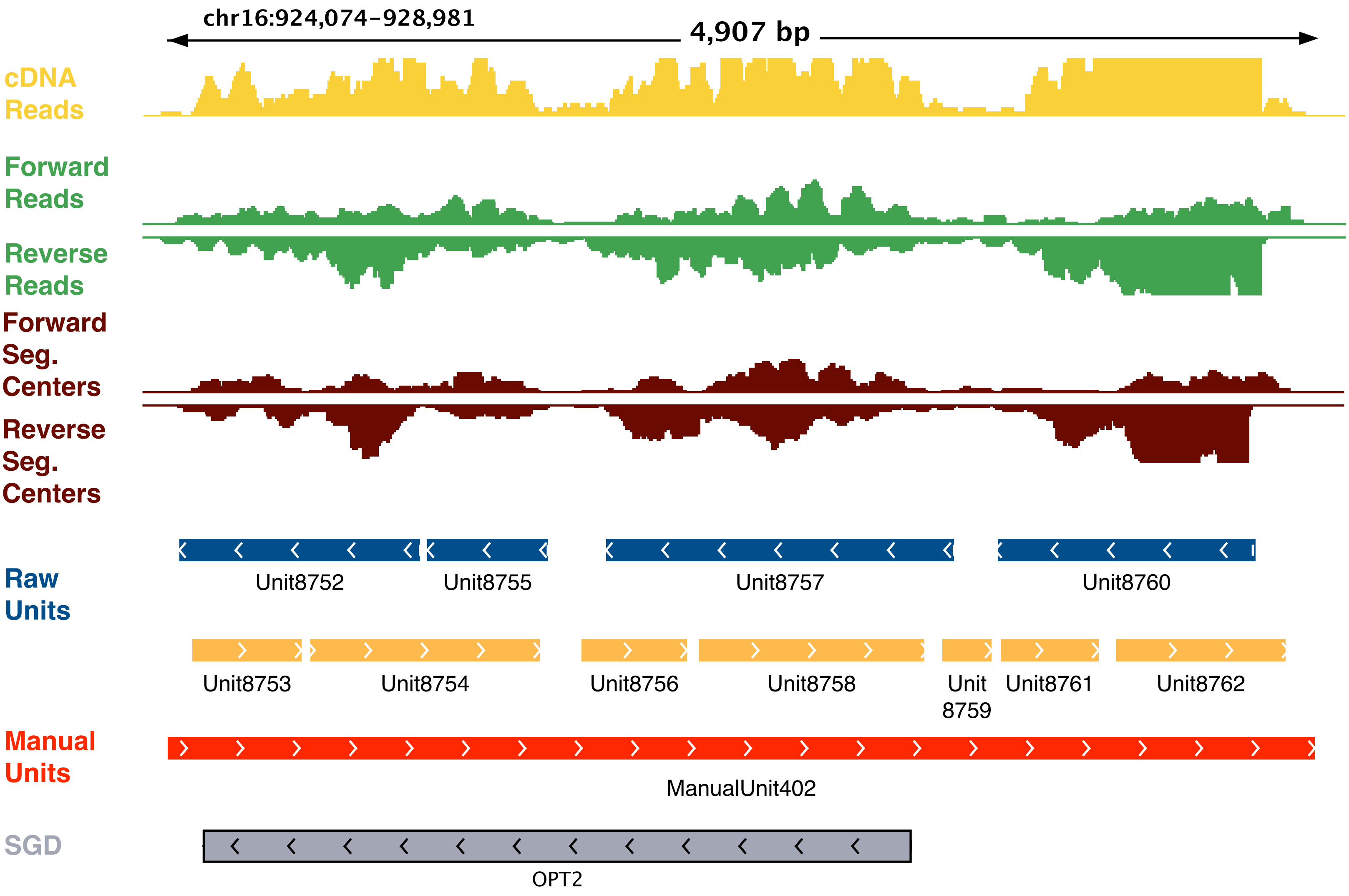

Supplementary Figure 4 - Antisense Units' Promoter Types

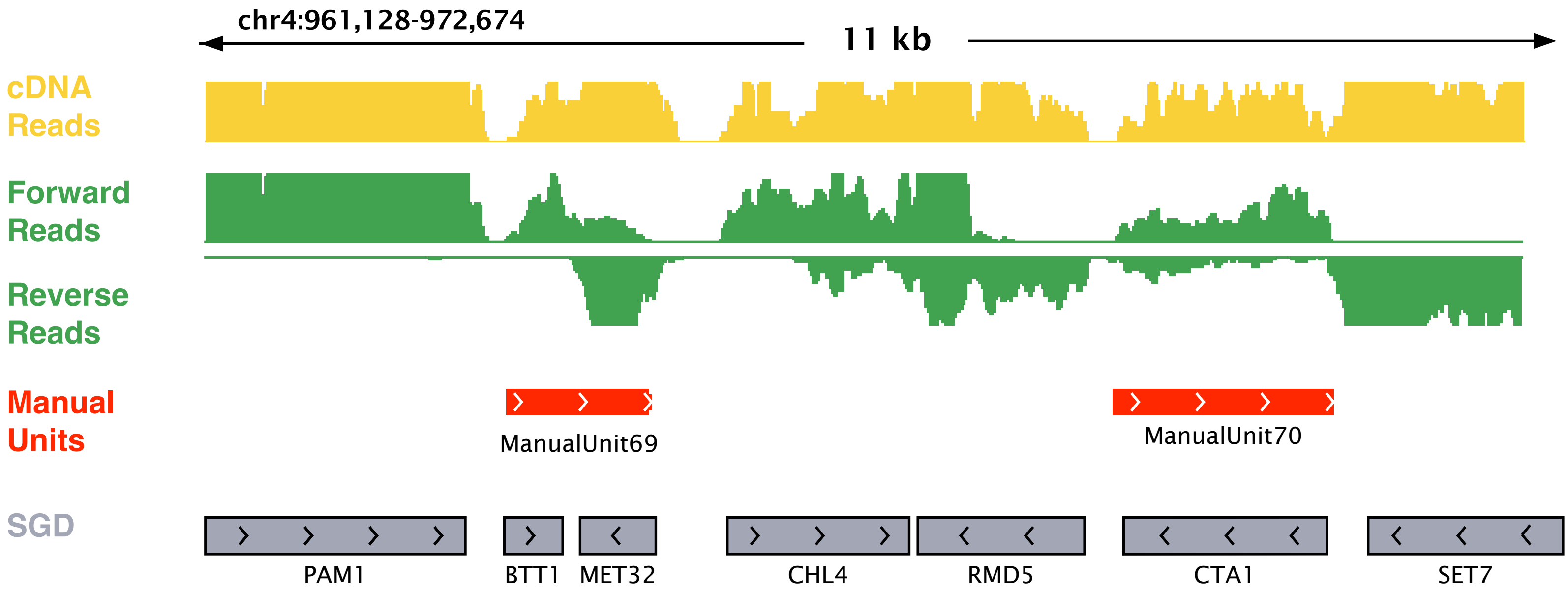

Supplementary Figure 5 - Expression patterns of antisense units and their neighboring genes

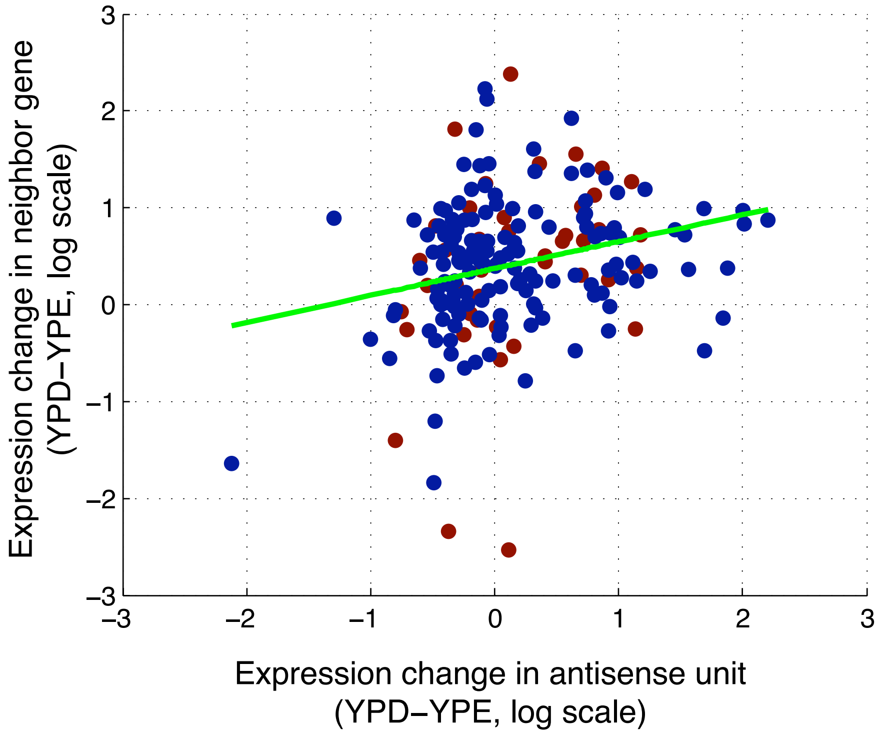

Supplementary Figure 6 - UTR length of genes with antisense ending close by

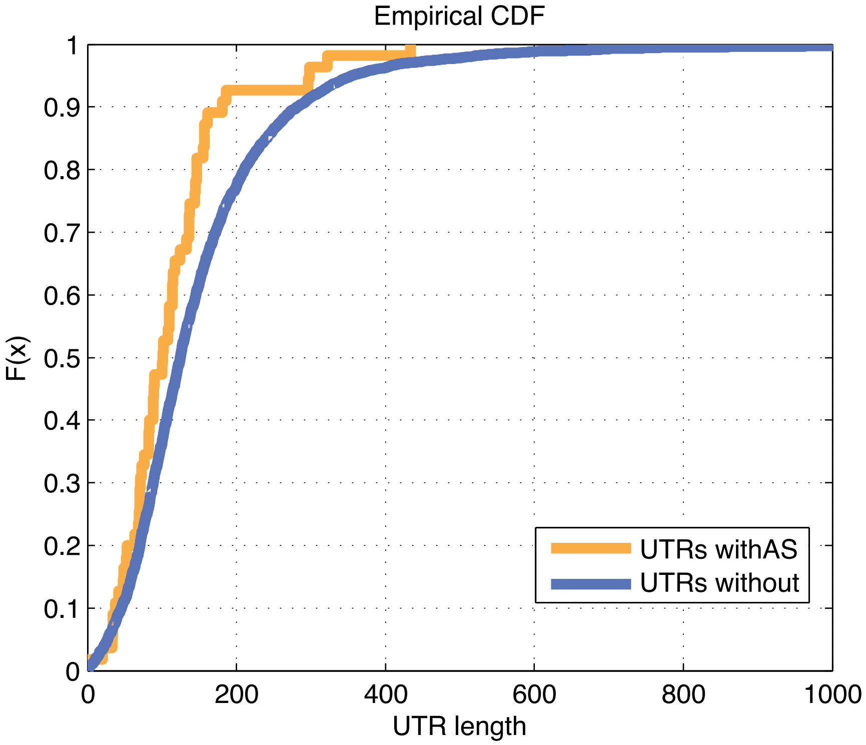

Supplementary Figure 7 - Expression Measurements

a Comparing YPE to YPD

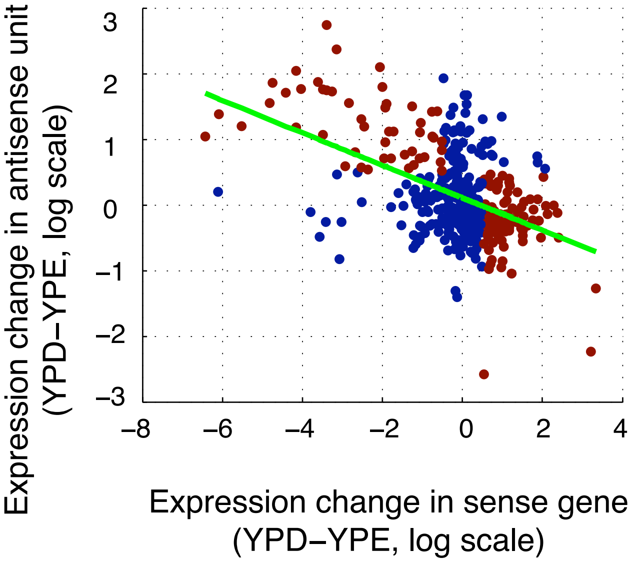

b Comparing YPGal to YPD

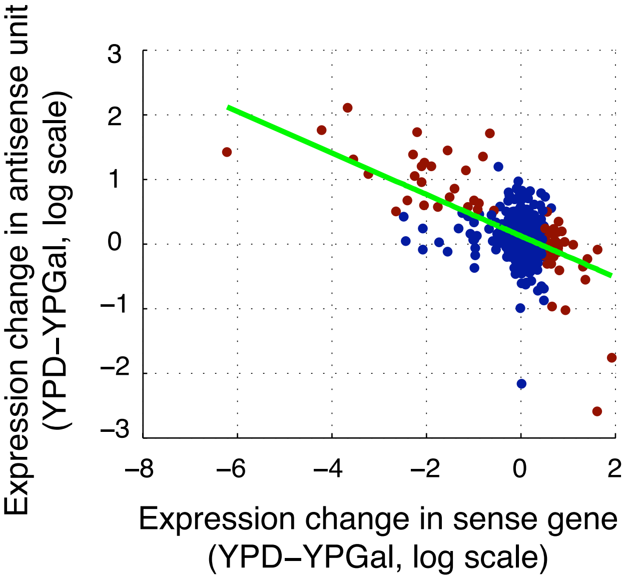

c Comparing Δrrp6 to YPD

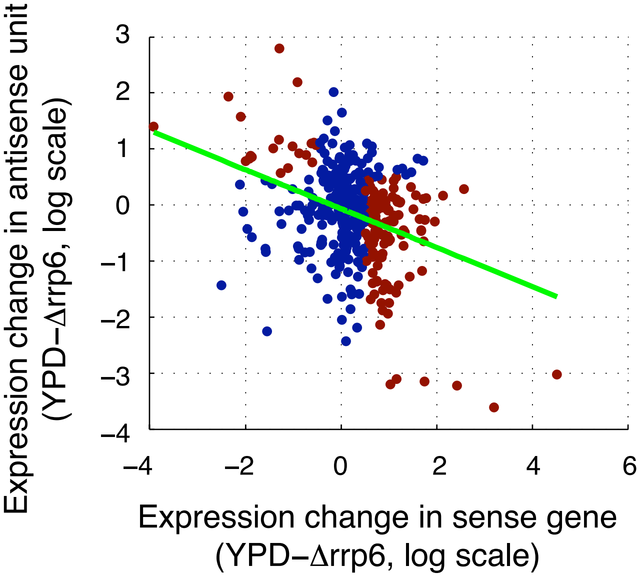

Supplementary Figure 8 - Mutant Effect on Transcription

a Δrrp6

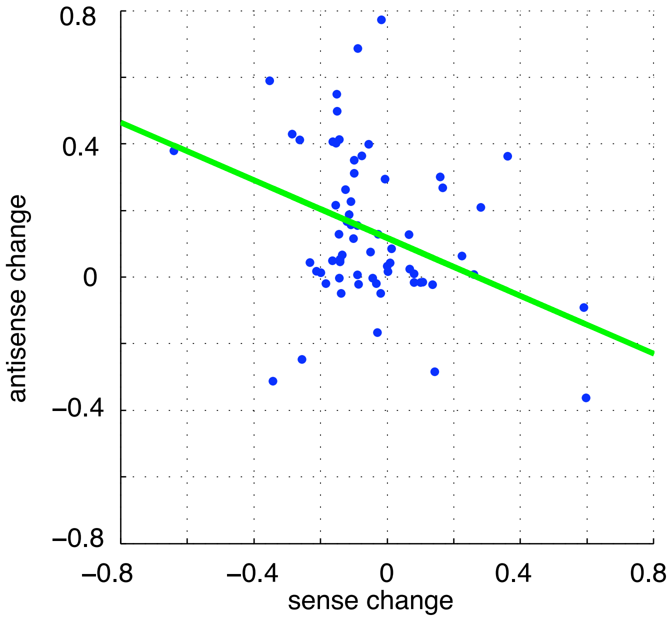

b Δhda2

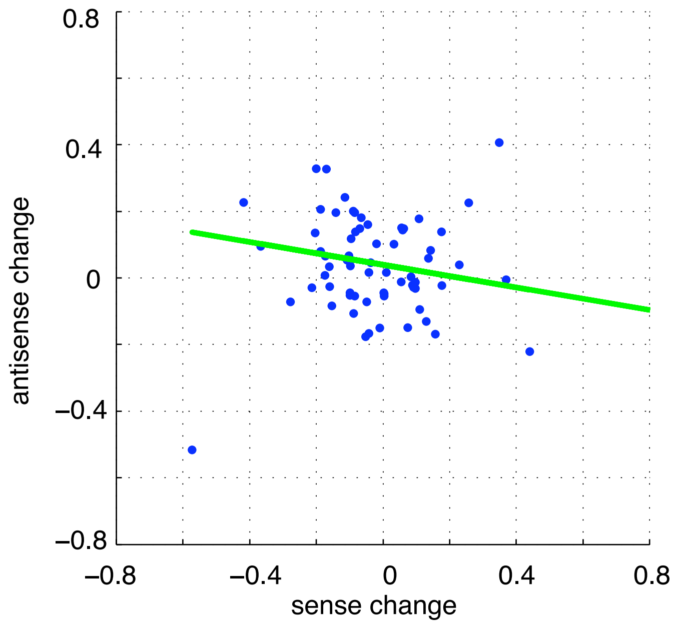

c Δrrp6Δhda2

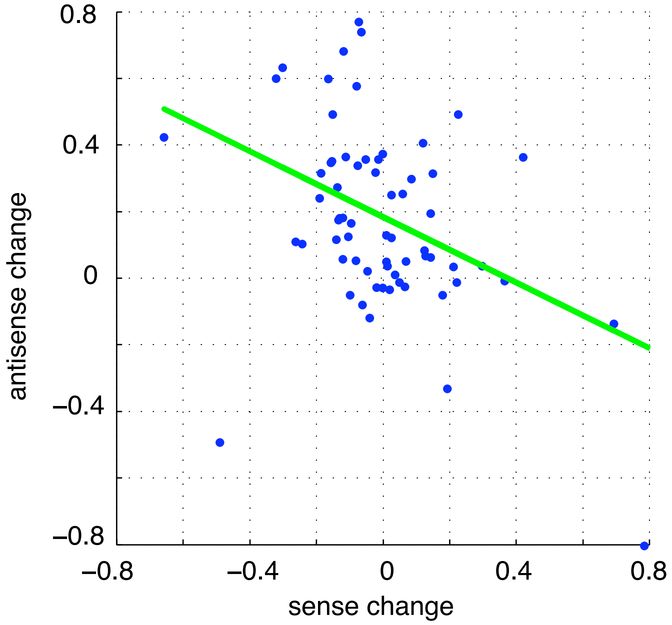

Supplementary Figure 9 - Mutant effect on sense differential expression

a Δrrp6

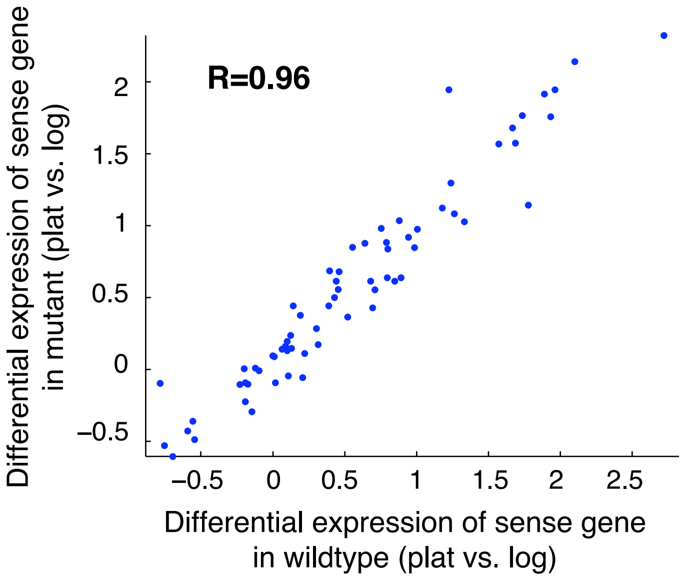

b Δhda2

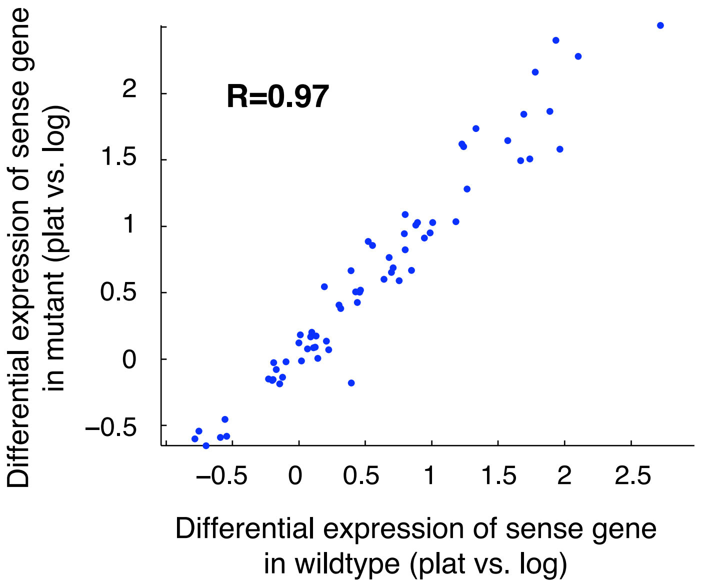

c Δrrp6Δhda2

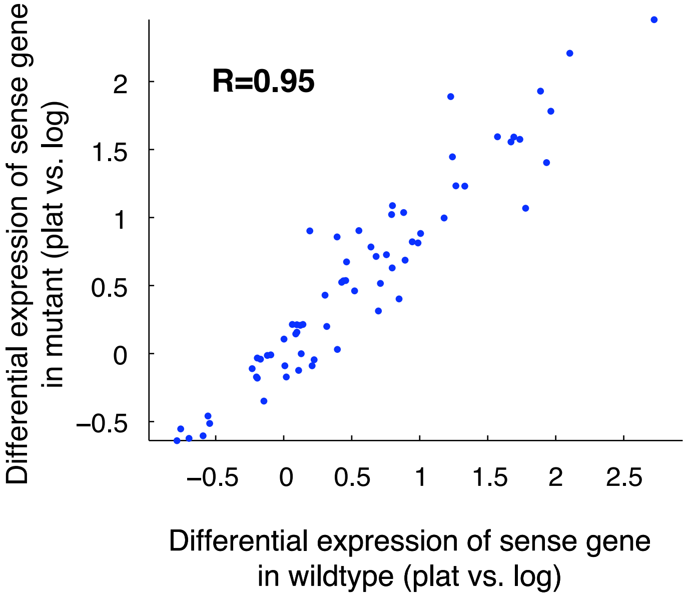

Supplement: Additional file 1 — Table S1. Strand-specific (sense and antisense) transcribed units in mid-log S. cerevisiae. [file gb-2010-11-8-r87-S1.PDF]
